# The Identification of Diabetes Mellitus Subtypes Applying Cluster Analysis Techniques: A Systematic Review

**DOI:** 10.3390/ijerph17249523

**Published:** 2020-12-18

**Authors:** Antonio Sarría-Santamera, Binur Orazumbekova, Tilektes Maulenkul, Abduzhappar Gaipov, Kuralay Atageldiyeva

**Affiliations:** 1 Department of Medicine, Nazarbayev University School of Medicine, Nur-Sultan 010000, Kazakhstan; binur.orazumbekova@nu.edu.kz (B.O.); tilektes.maulenkul@nu.edu.kz (T.M.); abduzhapar.gaipov@nu.edu.kz (A.G.); kurulay.atageldiyeva@nu.edu.kz (K.A.); 2 Spanish Network of Health Services Research and Chronic Diseases, REDISSEC, 28001 Madrid, Spain

**Keywords:** diabetes, novel sub-groups, unsupervised learning techniques, cluster analysis

## Abstract

Diabetes Mellitus is a chronic and lifelong disease that incurs a huge burden to healthcare systems. Its prevalence is on the rise worldwide. Diabetes is more complex than the classification of Type 1 and 2 may suggest. The purpose of this systematic review was to identify the research studies that tried to find new sub-groups of diabetes patients by using unsupervised learning methods. The search was conducted on Pubmed and Medline databases by two independent researchers. All time publications on cluster analysis of diabetes patients were selected and analysed. Among fourteen studies that were included in the final review, five studies found five identical clusters: Severe Autoimmune Diabetes; Severe Insulin-Deficient Diabetes; Severe Insulin-Resistant Diabetes; Mild Obesity-Related Diabetes; and Mild Age-Related Diabetes. In addition, two studies found the same clusters, except Severe Autoimmune Diabetes cluster. Results of other studies differed from one to another and were less consistent. Cluster analysis enabled finding non-classic heterogeneity in diabetes, but there is still a necessity to explore and validate the capabilities of cluster analysis in more diverse and wider populations.

## 1. Introduction

Diabetes Mellitus (DM) is a chronic and lifelong metabolic disorder characterized by elevated levels of glucose circulating in the blood that occurs either when the pancreas does not secrete enough insulin, due to destruction of pancreatic β-cells; when the body’s cells do not respond to insulin effectively; or by a combination of both mechanisms. The prevalence of DM has increased across the globe and is expected to rise to 592 million by 2035, incurring tremendous human, economic and social costs [1]. 

DM imposes a considerable burden on society in the form of low productivity, poor quality of life, increased healthcare expenditures, and premature mortality. The global cost of DM is overwhelming: US $1.31 trillion or 1.8% of global GDP. Notably, indirect costs accounted for 34.7% of the total burden [2].

DM significantly increases the risk of mortality: 1 in 12 of all-cause deaths may be attributable to DM [3,4,5]. Regardless of existence of effective treatments, DM outcomes are poor: DM patients show high frequency of serious and life-threatening micro- and macrovascular complications (strokes, acute coronary events, blindness, amputations, renal disease, heart failure) and premature mortality exceeding the general population [6].

DM management is challenging because of the heterogeneity in individual patient responses, which vary due to factors such as illness severity, sociodemographic characteristics, and specific clinical factors (e.g., glycated hemoglobin (HbA1c), insulin sensitivity, body composition, and duration of disease) [7]. DM is much more complex than the classification into Type 1 and Type 2 suggests. Recently, Alhqvist and colleagues using K-means cluster analysis (CA) has proposed a novel classification of adult onset DM into five subgroups: Severe Autoimmune Diabetes (SAID); Severe Insulin-Deficient Diabetes (SIDD); Severe Insulin-Resistant Diabetes (SIRD); Mild Obesity-Related Diabetes (MOD); and Mild Age-Related Diabetes (MARD) [7]. This classification is based on six measures that are commonly collected in clinical practice: body mass index (BMI); age at DM diagnosis; HbA1C; β-cell functioning; insulin resistance; and the presence of DM-related autoantibodies. The five subgroups differ in their patterns of progression and risk of complications. Currently, there is a rising interest in identifying more homogeneous groups of DM patients so therapeutic plans could be applied in a more targeted manner. New analytic techniques, namely unsupervised learning methods, such as CA, have been used in a variety of settings, with various sources and information and including different types of variables for proposing subtypes of DM patients.

The objective of this work is to systematically review the scientific literature to identify publications that have applied CA to generate homogeneous groups of DM patients, describe the main features of the analytic techniques that have been applied, as well as the variables included to propose DM subgroups.

## 2. Methods

### 2.1. Search Strategy and Selection Criteria

We systematically searched Medline Complete (from 1978 until August 2020) and PubMed (1974 until August 2020) databases on 7 August 2020 following PRISMA guidelines. Additionally, the reference lists of the selected articles from the above-mentioned databases were hand-searched. 

In the databases we searched studies published on the area of unsupervised CA of DM patients. The search strategy applying Medical Subject Headings (MeSH) was used in the Medline Complete database with the following keywords: “Diabetes Mellitus” or “Diabetes Mellitus, Type 2” or “Diabetes Mellitus, Type 1” or “Diabetes” AND “Cluster analysis” or “Cluster”. In the Pubmed database papers were searched applying “Diabetes” and “Cluster” keywords. The results were limited to articles in the English language and which had humans as a research subject. All database-specific technical variations were taken into account during the search. 

### 2.2. Methods of the Review

The selection process was performed by two independent researchers. Search results from two databases were combined to remove duplicates, after which all unique results were screened based on the title and abstract. In the next stage, full-text articles of potentially suitable articles were obtained and assessed for eligibility criteria: (1) the study population consisted of diabetic patients (Type 1 and/or Type 2 DM); (2) clusters were identified through one of the unsupervised clustering algorithms; (3) clustering was based on the patients’ clinical data. Studies with specific aims were excluded to provide comparability within clusters.

### 2.3. Data Extraction

The information was retrieved by two authors from selected articles to the a priori prepared tables, with the following columns: study design, source of the data taken for exploration, size and characteristics of targeted population, diagnostic criteria of DM, variables chosen for cluster analysis, and the number of clusters and their characteristics, as well as the data standardization, chosen clustering algorithm, methods for the determination on the number of clusters, and validation of clusters on an independent sample (please, see Appendix A and Appendix B). 

## 3. Results

**Figure 1:**
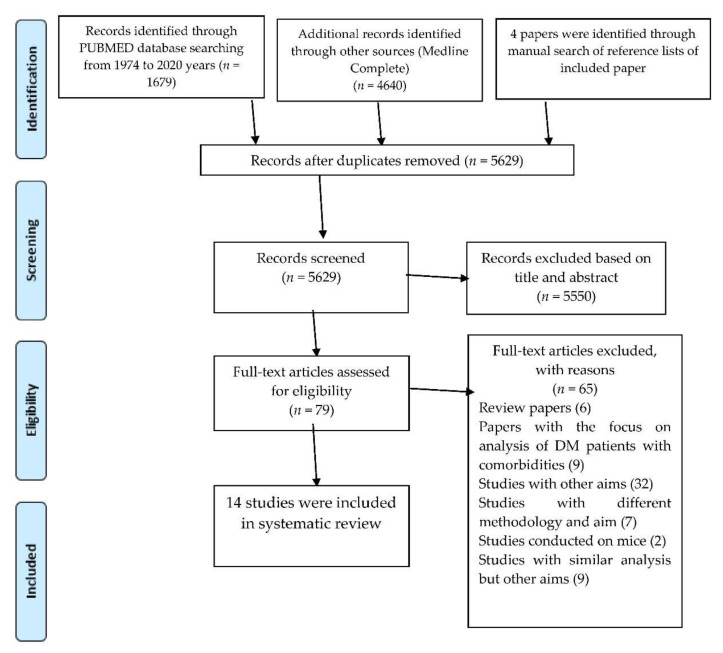
PRISMA Flow Diagram.

The search identified 6319 publications from two databases. After removing duplicates and screening the papers, 75 full-text articles were reviewed and 65 were excluded for the following reasons: 6 were review articles, 9 papers focused on exploring clusters of diabetic patients with specific comorbidities at baseline, 32 studies pursued other aims than finding subgroups of DM, 7 studies used other methodologies than unsupervised learning techniques, 9 studies conducted a similar analysis but with other specific aims (clustering of genetic data etc.), and 2 studies were conducted on mice. As a result, 14 papers were found to be eligible: 10 articles were included in the review [7,8,9,10,11,12,13,14,15,16] and an additional 4 eligible papers were found after hand-searching of the reference lists of selected articles [17,18,19,20]. The selection process is presented in Figure 1.

### 3.1. Sample Characteristics

The sample size ranged between 33 and 85,783 participants within studies constituting a total 130,353 diabetic patients: 33 type 1 diabetes (T1DM) patients, 238 latent autoimmune diabetes patients (LADA) and 130,082 type 2 DM (T2DM) patients. The largest sample size was in the study of Karpati et al. from Israel, constituting 85,783 patients of whom 60,423 were considered eligible for cluster analysis [15]. The second largest was the study of Kahkoska et al., with 20,274 DM patients [14], followed by 8980 individuals from the ANDIS cohort in the study of Ahlgvist et al. [7,20] The study with the smallest sample of 33 T1DM patients from several university hospitals was conducted in the UK [11]. 

The variability in population size could be explained by the source of the data, as data were taken from electronic medical records, healthcare databases, from previously conducted longitudinal observational studies and surveys. Disease duration among target populations of reviewed publications, along with newly diagnosed diabetic patients, ranged from 40 days after diagnosis to 12 years or longer [14,20]. The age of the participants varied depending on the type of DM: 5–16 years among T1DM patients, LADA patients were 35 years and older, the age of T2DM patients were between 18–96 years. Different criteria were used for the diagnosis of DM in the studies: American Diabetes Association Criteria [9], 1999 World Health Organization criteria [17], International Diabetes Federation diagnostic guidelines [12]. When data were extracted from health records or healthcare databases, diagnosis was based on specific ICD-10 codes for DM or antidiabetic medications [7,8,14,15,16,19,20]. Some studies used different diagnostic methods using biochemical indicators (fasting plasma glucose/HbA1c levels/blood test for autoimmune responses) with or without restrictions on the duration of treatment [10,11,18], while one study used self-reported DM cases [13].

The 14 selected studies, published between 2003 and 2020 years, were observational retrospective studies, 7 studies with follow-up periods [7,8,9,14,16,18,20] and 7 cross-sectional studies [10,11,12,13,15,17,19]. Reviewed studies were originated from different countries (Japan [8], Germany [9], USA [14,17,19], China [17], UK [11,18], Sweden [16,20], Italy [10,12], Israel [15], Australia [13] and Denmark [7]).

### 3.2. Cluster Analysis

#### 3.2.1. Data Standardization

Seven studies did not perform data standardization before doing the CA [8,9,11,12,13,15,17]. Two studies reported a presentation of the mean and standard deviation for values [10,19], three studies reported centering the values [14,18,20], and two studies reported calculating the mean of 0 standard deviation of 1 [7,16].

#### 3.2.2. Variables Selected for Cluster Analysis

Eight studies had almost similar variables for CA and the difference was trivial [7,8,9,14,16,17,18,20]. The following variables were, mainly, included in the CA of those eight studies: age at diagnosis; BMI; glutamic acid decarboxylase antibody (GADA) level; HbA1c level; homoeostasis model assessment 2 estimates of β-cell function (HOMA-2b); homoeostasis model assessment 2 estimates of insulin resistance (HOMA-IR).

Amato and colleagues used measurements of glucagon-like peptide-1 (GLP-1), glucose- dependent insulinotropic polypeptide (GIP), ghrelin for clustering [10].

Arif and colleagues included the following variables: interferon-g, interleukin 10 (Il-10), antigen-specific autoantibodies (Aabs), proinsulin, insulin, islet antigen antibodies (IA-2Ab), glutamic acid decarboxylase 65 antibody, zinc transporter 8 antibody [11].

Pes and colleagues also had quite distinct variables for clustering: gender, BMI, total cholesterol, triglycerides, systolic blood pressure, diastolic blood pressure, anti-glutamic acid decarboxylase (GAD) autoantibody, anti-islet antigen-2, anti-thyroid peroxidase, cumulative genetic score, insulin-free period [12].

Hammer and colleagues tried to cluster participants with DM according to self-reported symptoms, including, upper GI/dysmotility, diarrhea, constipation, nausea/vomiting [13].

Karpati and colleagues focused on clustering based on HbA1c levels. Thus, changes in HbA1c levels during the 3 year period, mean of the absolute first differences in HbA1c, and the ratio of the maximum absolute second difference to mean absolute first difference of HbA1c have been measured and included in the CA [15].

Li and colleagues had the highest number of variables included for CA among studies included in this systematic review, 73 variables [19].

Methods for determining the number of clusters varied from one study to another. Seven papers used the direct silhouette width method [7,8,9,14,17,18,20], one paper had a fixed number of clusters [10], one study determined the number of clusters based on hierarchical clustering with Ward’s method [11]. In addition, two publications determined the number of clusters based on principal component analysis (PCA) [12,13], one publication performed a “NbClust” algorithm that selected an optimal method for the determination of number of clusters [15], one study was based within the cluster sums of squares against the number of clusters [16], one study was based on a cosine distance metric [19].

#### 3.2.3. Methods of Clustering and Dimensionality Reduction

Only two studies have indicated reducing the dimensionality of the data prior to CA [12,13].

The widespread method for clustering among included publications was k-means clustering [7,8,13,15,16,17,18,20]. Several studies performed k-means analysis only for GADA-negative individuals [7,8,18]. The second widespread method of clustering was hierarchical CA: six studies reported performing hierarchical clustering [7,8,9,10,11,14]. The least frequent methods for clustering were PCA [12,16] and topology-based analysis (TBA) [19].

#### 3.2.4. Cluster Validation on an Independent Sample

Only five studies performed validation of results of CA on an independent sample [7,18,19,20], while Karpati et al. split the database to train and test datasets to replicate findings [15].

#### 3.2.5. Main Results

Two [10,11], three [15,19], four [12,13,14,17] and five [7,8,9,16,18,20] different clusters were identified in the reviewed papers. The majority of studies revealed the same 5 clusters: SAID, SIDD, SIRD, MOD, and MARD [7,8,9,18,20]. Two additional studies identified the same four clusters except SAID, due to the unavailability of GADA measurements [14,17]. The proportion of SAID cluster varied between 4% and 22.3% in the studies with the same applied cluster name, while the Autoimmune β-cell failure cluster described by Safai et al. was identical to SAID with GAD-positive antibodies comprising 2.8% of the total sample [7,8,9,16,18,20]. The proportion of the SIDD cluster was between 2.5% and 20% within studies, while the non-autoimmune β-cell failure cluster identified by Safai et al. shared similar characteristics to SIDD and composed 22.3% of the total sample [7,8,9,14,16,17,18,20]. The proportion of the SIRD cluster ranged within 7.2% and 23.7% among studies, while 2 similar clusters were revealed by Safai et al. such as insulin resistance with short disease duration (21.4%) and insulin resistance with long disease duration (31.7%) [7,8,9,14,16,17,18,20]. The next most frequent cluster was MOD with varying percentages between studies from 20.4% to 34% [7,8,9,14,17,18,20]. The MARD cluster was the most prevalent among the mentioned five clusters in each study, falling within 34% and 45.4% [7,8,9,14,17,18,20]. Additionally, Safai et al. reported a cluster based on the presence of metabolic syndrome, which had the highest BMI and constituted 21.7%, but differed by clinical characteristics from the aforementioned MOD and MARD clusters [16].

The main five clusters identified across studies shared similar phenotypic characteristics. All of the patients in the first SAID cluster were GADA-positive, were younger compared to other cluster members, had low BMI and insulin deficiency characterized by low HOMA-2b and higher HbA1c levels. The patients with DM in the SIDD cluster had the same characteristics but were GADA-negative. At the same time, participants from SIRD differed with high BMI, whole-body and/or adipose-tissue insulin resistance characterized by high HOMA-IR and were at a relatively younger age. Individuals in the MOD cluster were slightly younger and had obesity and moderate insulin resistance compared to the SIRD cluster. The oldest age of diabetic patients and moderate metabolic dysregulations were inherent to the MARD cluster. Authors in the reviewed papers identified several complications associated with each cluster, which were also observed in the replicated studies. The major conditions were diabetic or chronic kidney diseases (DKD, CKD), liver diseases (non-alcoholic fatty liver disease (NAFLD) or hepatic fibrosis), retinopathy, polyneuropathies, and cardiovascular diseases (CVDs). Thus, in studies of Zaharia et al. and Ahlgvist et al., the SIRD cluster and in the study of Tanabe et al., both SIRD and SAID clusters were associated with a higher risk for CKD and DKD [7,8,9,20]. The cluster with the presence of metabolic syndrome in the study conducted by Safai et al. reported the same association with nephropathies [16]. However, Dennis et al. did not find an increased risk for CKD complications among clusters after adjustment for baseline estimated glomerular filtration rate (eGFR) [18]. SAID and SIDD in the study of Tanabe et al., but only the SIDD cluster in the studies of Ahlgvist et al., were associated with the increased risk for retinopathy [7,8,20]. Along with them, the similar non-autoimmune b-cell failure cluster to SIDD in the study of Safai et al. demonstrated the same association with retinopathy [16]. Liver diseases such as NAFLD and hepatic fibrosis were found to be associated with the SIRD cluster in studies of Zaharia et al. and both studies of Ahlgvist et al. [7,9,20] At the same time, neuropathies identified in the Zaharia et al. study among SIDD individuals, were not associated with any cluster after adjustment for disease duration or age at onset in the study of Safai et al. [9,16] In the study of Kahkoska et al., unadjusted analysis showed that CVDs were associated with the SIDD cluster, which is characterized by low BMI and insulin deficiency [14]. However, CVDs did not differ among clusters after adjustment for known modifiable and non-modifiable risk factors in the studies of Safai et al. and Tanabe et al. [8,16]

Amato et al. phenotyped diabetic patients based on fasting incretin levels into two independent clusters: cluster 1 (65.6%) with lower incretin levels and cluster 2 (34.4%) with higher incretin levels [10]. Thus, cluster 1 differed by a lower glucagon-like peptide-1 (GLP-1), glucose-dependent insulinotropic polypeptide (GIP) and, consequently, with higher levels of HbA1c and fasting plasma glucose (FPG) compared to cluster 2, which was explained by possible increased a-cell activity and its effect on the reduction in b-cell function. However, there were no differences in the clinical-anthropometric characteristics between clusters.

Based on the data from electronic medical records, Li et al. clustered T2DM patients applying TBA and came up with three different subtypes with inherent clinical characteristics and comorbidities [19]. Individuals in subtype 1 had higher weight and serum glucose levels and were associated with diabetic nephropathy and retinopathies, patients in subtype 2 had lower weight and were associated with cancer malignancy and CVDs, while subtype 3 was characterized by neurological diseases, allergies, HIV and CVDs.

Karpati et al. found ascending (14.4%, mean HbA1c 8.7% (1.9)), descending (10.0%, mean HbA1c 7.8% (1.8)) and stable (75.6%, mean HbA1c 7.1% (1.2)) subtypes of T2DM patients, with the duration of 3–7 years, based on their HbA1c levels’ trajectories and their five-year risk of complications [15]. Diabetic patients in the ascending cluster were the youngest compared to the representatives of other clusters, and were taking mostly non-insulin medications, while insulin medications were often prescribed to patients in the descending cluster. However, micro- and macrovascular complications were prevalent in both ascending and descending clusters. The mortality rate was higher in the descending cluster.

Hammer et al., based on gastro-intestinal symptoms of T2DM patients, found four4 such clusters as Upper GI/Dysmotility (44.8% of the total variance), Diarrhea (10.4% of the total variance), Constipation (7.8% of the total variance), and Nausea/Vomiting (6.3% of the total variance) [13]. Analysis in the given study has shown that oral medications taken by diabetic patients were associated with the Nausea/Vomiting cluster. After adjustment for the type of treatment (insulin or oral medication), gender, and age, members of Upper GI/Dysmotility cluster were heavily linked with use of insulin in conjunction with hypoglycemic medication, while Nausea/Vomiting cluster members had a strong relationship with the intake of insulin, oral hypoglycemic medication, and with the combination of both. Diarrhea and Constipation clusters have not shown any significant linkages.

Arif et al. found two clusters of T1DM patients by assessment of different parameters of autoimmunity of CD4 T-cell and B-lymphocyte responses [11]. Thus, T1DM patients in the later stages are differentiated with (AAb++ and IFN-g. IL-10) and (AAb6 and IFN-g, IL-10), as well as other non-diabetic individuals with high AAbs who had an increased risk for T1DM development. Overall, cluster 1 was dominated for IL-10 response to GAD, insulin, and proinsulin compared to cluster 2.

Pes et al. found four different clusters of LADA patients. Each cluster had a special set of important characteristics extracted based on the PCA. One of the main findings related to the disease progression was the association of b-cell function with four clusters (PCs) [12]. The fastest b-cell failure was observed among members of PC 2, which was characterized by genetic profile, while mild and slower b-cell activity was seen among PC 1, as well as gender and TGs predominated PC 3 with cholesterol predominated PC 4, respectively.

## 4. Discussion

The main finding of this systematic review is that data-driven algorithms reflect a larger heterogeneity in DM subtypes that the classical division into T1DM and T2DM or solely based on glycemic or HbA1c levels may reflect. Another finding is that a significant number of studies with data from a diversity of patient origins receiving the same five clusters of DM patients, which shared similar physiological and clinical characteristics across studies and were associated at most with analogous comorbidities, although having a different prevalence as well as variations across them in the frequency of the variables included in each of them. However, there were also six papers that provided clusters of DM patients based on different types of variables shown also to be appropriate in terms of statistical significance as well as clinical meaning. Another relevant finding is that there is significant variability in terms of the use of specific analytic techniques to generate those clusters of DM patients.

Overall, those findings confirm that the process of using clustering techniques, although not exempt from certain limitations, may be applied for monitoring the progression and control of patients with DM, but there is still uncertainty on the variables that should be used for generating subtypes of patients, as well as for what is the most appropriate clustering method.

As for the studies that proposed the same five clusters, the proportions of individuals in each cluster varied from one study to another. Several factors may influence those disproportionate distributions. First, the source of the data applied for CA in the studies varied based on the availability and may explain some variations in the sample size, as well as the type of diabetic patients participating in the analysis. For instance, the extreme proportion of SAID patients in the study of Zaharia et al. could be explained by active recruitment of T1DM patients, while studies that have utilized data from other cohorts showed consistent results [9]. Second, some cohorts used for CA were focused specifically on the studies with DM patients at onset [7,9,10,11,17,20], while others recruited patients with a longer [8,13,14,15,16,18] or not defined [12,19] duration of the disease. Thus, characteristics of DM patients with a longer duration of the disease may overlap with other comorbid conditions, consequently making it difficult to differentiate specific characteristics inherent to each cluster. Along with this, medications or lifestyle factors of those patients may shade the real trajectories of the disease progression, as Zaharia et al. demonstrated redistribution of 23% of all members in clusters during a 5-year follow-up period [9].

The effect of ethnicity on the clustering results is still an open question, as most of the studies were limited to the representatives of one ethnic group, except Ahlgvist et al., Zou et al. and Kahkoska et al., who validated their results on the databases originated from diverse geographical locations [7,14,17]. This is an important aspect, since the clustering results of the Japanese population showed that Asian diabetic patients, due to their inherent lower b-cell activity and insulin secretion, showed a higher proportion of SIRD cluster with a comparatively lower BMI than the studies from western cohorts, meaning there is a potential earlier onset of DM in their population [7,8,14,21].

Overall, five main clusters were reproducible in the studies which used databases from cross-sectional, longitudinal observational and trial studies. All the aforementioned papers, revealing meaningful complications specific for clusters, used data from longitudinal observational studies. The cross-sectional study of Zou et al. and of Kahkoska et al., which selected patients with a baseline high risk for CVDs and long-lasting DM, were not able to estimate risks for complications [14,17], while Dennis et al., who did the study with protocol-driven follow-up, were able to find out several complications adjusting to different treatments [18].

The range of associated comorbidities is not limited to the aforementioned conditions. There might be other complications of diabetic patients that would eventually need to be considered in the further clustering studies. Li et al., in their study, observed a wider range of associated comorbidities applying TBA [19]. Adjustment for known modifiable and non-modifiable risk factors are also suggested to determine their true effect, as some studies showed no association with CVDs, indicating the importance of sticking to a healthy lifestyle to reduce the risk of complications [8,16].

Another relevant issue still in need of further investigation is the optimal number of variables which provide the balance between validity and economic efficiency of clustering diabetic patients: Kahkoska et al., using only three variables (age, BMI, and HbA1c), obtained the four clusters with very similar characteristics to the original clusters proposed by Ahlgvist et al. with six variables.

Other studies which found clusters of diabetic patients with different GI symptoms [13], fasting incretin tone [10], trajectories of HbA1c levels [15], clusters among T1DM [11] and LADA [12] patients, as well as clusters identified through novel TBA [19], were unique and not replicated and therefore should be considered as a call for future research initiatives.

However, the study of Karpati et al., with a sufficient sample size of 60,423 patients, identified interesting findings by clustering based on HbA1c levels: the ascending cluster had complications only in the extremely high levels, which could possibly suggest other risk factors among this group, while the highest risk for complications among DM patients were found in the stable cluster with HbA1c < 6.0%, which contradicts the guideline recommendations and is consistent with J-shaped risk [15,22].

Moreover, the only study of clustering T1DM identified patients with different immunological responses and could be implicated in the clinical practice by tailoring immune-based therapies, raising issues about the underlying basis for the different phenotypes observed if they reflect the different immunological pathways of the disease.

Overall, results of all studies indicated the need to pay attention to symptoms and clinical characteristics of the diabetic patients, which previously were underestimated and may have an impact on their disease progression, as well as on the need to incorporate the wealth of information of unstructured data from the free text of patient records [23]. Genetic information is another critical domain that will be necessary to explore in order to identify subgroups of DM patients [24].

Review studies applied different methodological approaches of CA. Each step before and during the running CA in different ways may affect the clustering outputs. It is critical not to violate the reproducibility of unsupervised learning techniques, therefore, validation in different datasets is required to provide robustness of the results. Second, the type of data (observational/longitudinal) is also critical in cluster analysis to give a chance to observe temporal patterns of disease progression, as cluster analysis does not explain the aetiology of the disease. Third, the number of clusters depends on the specific methodology applied as well as the proportions of populations among clusters that could vary based on the chosen sample size and the presence/absence of scaling the dataset (preprocessing) [25].

Among the issues related to methods for determining the number of clusters, one study has chosen to limit the number of clusters to two [10]. Manually limiting the number of clusters could lead to error as there might be more clusters within the data.

Regarding the methods for clustering, seven out of fourteen studies have performed a k-means clustering. Several studies relied solely on k-means, other studies have performed it only to confirm the results from the hierarchical clustering or to cluster only GADA-negative individuals. In k-means clusters, the presence of outliers could distort the results of clusterization [26]. Among seven studies, only two reported excluding outliers prior to clustering [18,20]. Performing k-means requires running the clustering multiple times to obtain optimal results, but it also increases the risk of ending in a local optimum. The local optima is characterized by poorer quality of clusters that might affect the number of clusters [27]. None of the studies reported minimizing the local optima. The next widespread method after k-means was hierarchical clustering. The distance metric and linkage criteria choices ranged among six studies that performed hierarchical clustering. Those choices could affect the result of clustering as, currently, there is no sturdy theoretical justification for such decisions. Another issue with hierarchical clustering is the treatment of missing values. Most software does not work if this is the case. Four studies have not reported the presence or absence of missing data variables [7,9,10,14]. The third widespread method for clustering was PCA [12,16]. Pes and colleagues have not reported standardizing the data standardization prior to PCA, which is essential to enable the PCA with the search of optimal principal components [12]. The last method to discuss is TBA. Li and colleagues performed TBA, which is quite new in machine learning and it has a strong theoretical basis [19].

The next aspect to discuss is the validation of clustering results. Nine studies have not reported validating clustering results [8,9,10,11,12,13,14,16,17]. The validation of the results by external validation on an independent sample or cross-validation within a dataset is vital to obtain the information on the quality of performed CA [28].

The data standardization process is also an important step to enable comparison of variables that could have units at different scales. Without standardization, variables with different scales would unequally contribute to the results of analysis [29]. Only seven studies out of fourteen have reported standardizing the data prior to CA.

Some common limitations among the included studies were: the lack of some variables in their data that would affect the clustering results [7,12,16,17,18,20]; having small or relatively small sample sizes for doing clustering [8,10,11,19]; issues that may affect the generalizability of the results [8,9,14]; and having a relatively short follow-up of participants [11,12,13,14,15]. Last but not least, Hammer et al. had reported grouping all oral medications into one group, while some drugs, such as metformin, could have significantly different effects on controlling the high blood sugar than other drugs [13]. Thus, it might have affected the results of clustering.

## 5. Conclusions

This systematic review has explored the research publications that utilized clustering algorithms to identify non-classic heterogeneity in DM. DM is a complex condition and clustering analysis is showing to be an effective method for finding clinically meaningful subgroups. Identifying homogeneous subgroups of patients with potential disease progression at an onset, based on routinely collected measurements, could be useful to apply therapeutic and prevention measures, targeting patients that will be benefitted the most. There is a significant number of effective therapeutic alternatives to treat DM, including insulin and oral medications, the latter having quite diverse mechanisms of action. It will be necessary to identify which sub-groups of patients with DM benefit most of those available therapies and advance towards more targeted treatments. Nevertheless, there are still some methodological aspects that must be clarified as well as what may be the metabolic pathways affected in each subgroup of patients. There is also a need for studies that would explore and validate the capabilities of CA in more diverse and wider populations, combining variables that have already shown statistical and clinical relevance to generate homogeneous groups of DM patients.

## Appendix A

**Table A1:** Characteristics of included studies.

	Study (Author, Year)	Country	Study Design	Source of the Data	Population Size and Characteristics	Diagnostic Criteria of Diabetes	Variables for Cluster Analysis	Number of Clusters and Characteristics
1.	Ahlqvist et al. (2018) [7]	Denmark	Observational retrospective study	Steno Diabetes Center Copenhagen database	N = 2290. The majority were Caucasians. There were more male smokers and ex-smokers. Males had a higher level of HbA1c, BP, weight and TG but lower BMI and cholesterol levels at baseline.	Health records with patients who had clinically diagnosed type 2 diabetes.	1. HbA1c 2. Age at diagnosis 3. Diabetes duration 4. BMI 5. HOMA2-IR 6. HOMA2-β 7. GAD65 autoantibody titre.	*Cluster 1* (SAID, *n* = 577): characterized by early-onset disease, relatively low BMI, poor metabolic control, insulin deficiency, and presence of GADA; *Cluster 2* (SIDD, *n* = 1575): GADA negative but otherwise similar to cluster 1: low age at onset, relatively low BMI, low insulin secretion (low HOMA2-B index), and poor metabolic control. *Cluster 3* (SIRD, *n* = 1373): characterized by insulin resistance (high HOMA2-IR index) and high BMI. *Cluster 4* (MOD, *n* = 1942): characterized by obesity but not by insulin resistance. *Cluster 5* (MARD, *n* = 3513): similar to cluster 4, only modest metabolic derangements.
2.	Tanabe et al. (2020) [8]	Japan	Observational retrospective study	Fukushima chronic kidney disease(CKD)cohort (January 2003–March 2017) and Fukushima Diabetes, Endocrinology and Metabolism(DEM)cohort (January 2003–November 2019)	1255 of 1520 (917 patients from CKD cohort and 603 from DEM cohort) T2DM patients included in cluster analysis	ICD-10 codes E10–14 or FPG ≥ 126 mg/dL, RPG ≥ 200 mg/dL, in a patient with classic symptoms of hyperglycemia or hyperglycemic crisis A1c ≥ 6.5%	1.GADA levels; 2.Age at diagnosis; 3.BMI; 4.HbA1c; 5. HOMA2-B; 6.HOMA2-IR	*cluster 1* (SAID, 68 (5.4%)): was positive for islet-related autoantibodies and was young at onset, had an increased risk of diabetic retinopathy, after adjusting for modifiable risk factors; *cluster 2* (SIDD, 238 (19.0%)): had a severe insulin deficiency and the highest A1c; *cluster 3* (SIRD, 90 (7.2%)): was the highest in BMI, HOMA 2-IR, and HOMA2-B and had an increased risk of DKD; *cluster 4* (MOD, 363 (28.9%)):had a higher BMI and was slightly younger than the MARD subgroup; *cluster 5* (MARD, 496 (39.5%).
3.	Zaharia et al. (2019) [9]	Germany	Observational retrospective study	T1DM and T2DM diabetes patients from prospective German Diabetes Study (01/2009 and 1/2015)	1105 patients with known disease duration of less than 12 months, aged 18–69 years	American Diabetes Association criteria	1. Age; 2. BMI; 3.Glycaemia, 4. HOMA-IR; 5. HOMA-B; 6. GADA levels.	*cluster 1* SAID (N = 247): GADA positive, were more likely to be of a younger age, had relatively low BMI, poor glycemic control and overt insulin deficiency. 158 (67.0%) received insulin on diagnosis *cluster 2* SIDD (N = 28): showed similarities with patients with SAID, but GADA negative; had the highest prevalence of confirmed diabetic sensorimotor polyneuropathy and cardiac autonomic neuropathy; 12 (44.0%) were treated with insulin on diagnosis; *cluster 3* SIRD (N = 121): had high BMI and whole-body adipose-tissue insulin resistance, had the highest sensitivity for C-reactive protein, high hepatocellular lipid content and fatty liver index, low eGFR levels; *cluster 4* MOD (N = 323): had obesity and substantial adipose tissue insulin resistance, high sensitivity for C-reactive protein, but they had moderate whole-body insulin resistance; *cluster 5* MARD (N = 386): older than those in other clusters and showed only minor metabolic abnormalities.
4.	Amato et al. (2016) [10]	Italy	Cross-sectional study	Outpatient clinic at Unit of Endocrinology, Diabetology and Metabolism, University of Palermo	N = 96. Caucasian patients with type 2 diabetes within 6 months of onset, age range 51–75 years.	Health records about known type 2 diabetes for <6 months and in stable treatment for the last 3 months with metformin	(1) glucagon-like peptide-1 (GLP-1) (2) glucose- dependent insulinotropic polypeptide (GIP) (3) ghrelin	*Cluster 1* (*n* = 63): significantly lower levels of GLP-1, GIP and ghrelin compared to *cluster 2* (*n* = 33), and higher levels of HbA1c and fasting plasma glucose. Regarding the clinical and anamnestic characteristics of the patients, there were not any significant differences between the two clusters, except for a greater prevalence of patients practicing physical activity in *Cluster 2*.
5.	Arif et al. (2014) [11]	UK	Cross-sectional study	Several university and regional hospitals in UK took part in the research	N = 33. Children with newly diagnosed type 1 diabetes (5–16 years), unaffected siblings of patients with type 1 diabetes (6–16 years).	Test of blood autoimmune response phenotypes by combinatorial, multiparameter analysis of autoantibodies and autoreactive T-cell responses	For Autoimmune Inflammatory Phenotypes in Children With Newly Diagnosed Type 1 Diabetes group: 1. interferon-g 2. interleukin 10 (Il-10) 3. antigen-specific autoantibodies (Aabs) 4. proinsulin 5. insulin 6. Islet antigen antibodies (IA-2Ab) 7. GAD65 antibody 8. zinc transporter 8 antibody	*Cluster 1* (*n* = 15): a combination of islet AAbs and IFN-g responses to all antigens. Have a significantly higher frequency of IL-10 response to GAD, insulin, proinsulin. There are also differences in the frequency of islet AAbs between clusters. AAbs against IA-2 and ZnT8 are significantly less frequent in the IL10–dominated cluster-1. Two children had no islet AAbs present at diagnosis, five had only a single AAb, and eight had two or more AAbs. *Cluster 2* (*n* = 18): The frequency of multiple AAbs was significantly higher, all 18 children had two or more IL-10 responses to all antigens.
6.	Pes et al. (2016) [12]	Italy	Cross-sectional study	Diabetic Unit, Department of Internal Medicine, University of Sassari, November 2005–December 2010	N = 238. Patients with a Latent autoimmune diabetes in adults. Patients were of Sardinian origin for at least 2 generations, with 35 and older age.	International Diabetes Federation worldwide consensus	1. Gender 2. Body mass index 3. Total cholesterol 4. Triglycerides 5. Sistolic blood pressure 6. Diastolic blood pressure 7. anti-glutamic acid decarboxylase (GAD) autoantibody 8.Anti-Islet Antigen-2 9. Anti-thyroid peroxidase 10. Cumulative genetic score 11. Insulin-free period	*PC 1* (explained 18.0% of total variance): the dominant variables were: BMI, triglycerides, systolic and diastolic blood pressure and duration of insulin-free time period, showed a mild beta-cells failure. *PC 2* (explained 15.0% of total variance): genetic variables such as Class II HLA, CTLA-4 as well as anti-GAD65, anti-IA-2 and anti-TPO antibody titers, and the insulin-free time period predominated, showed a faster beta-cells failure. *PC 3* (explained 12.0% of total variance): gender and triglycerides predominated, showed a slower beta-cells failure. *PC 4* (explained 12.0% of total variance): cholesterol predominated, showed a slower beta-cells failure.
7.	Hammer et al. (2003) [13]	Australia	Cross-sectional study	Survey	396 T2DM patients from 8555 surveyed. Two groups of the population of western Sydney. With diabetes (mean age 59.5 years), and without (44.6 years).	Self-reported	Self-reported symptoms: *Upper GI/Dysmotility* 1. Bloating 2. Food staying in stomach 3. Pain 4. Heartburn 5. Early satiety 6. Dysphagia *Diarrhea* 7. Urgency 8. Loose/watery stools 9. Less than 3 bowel movement/day 10. Fecal incontinence *Constipation* 11. Hard/lumpy stools 12. Blockage in the anus 13. Less than 3 bowel movement/week 14. Constipation/diarrhea *Nausea/Vomiting* 15. Vomiting 16. Nausea	The cluster analysis of the four latent symptom factors produced a five-cluster solution: Health group (5205) and four diseased clusters (396). The disease clusters were each defined by higher-than-average scores on a single symptom and were labeled according to that symptom. 1. Health. 2. Upper GI/Dysmotility (44.8% of the total variance): Poor glycemic control increased threefold compared to Health cluster. 3. Diarrhea (10.4% of the total variance)a: Poor glycemic control increased sevenfold compared to the Health cluster. 4. Constipation (7.8% of the total variance): Poor glycemic control increased fivefold compared to Health cluster. 5. Nausea/Vomiting (6·3% of the total variance): Poor glycemic control increased sixfold compared to Health cluster.
8.	Kahkoska et al. (2020) [14]	USA, Denmark, Germany	Observational retrospective study	Cardiovascular Outcome Trials’ data	N = 20,274. Participants recently enrolled from three randomized, double blind, controlled, parallel-group multinational CVOTs in adults with long-standing T1 and T2 diabetes. The mean age was 64 years or older and the mean duration of diabetes was 12 years or longer.	DEVOTE: if patients got treatment against diabetes LEADER, SUSTAIN: glycated hemoglobin level of 7.0% or more.	1.HbA1c 2.BMI at baseline, 3.Age at T2DM diagnosis	*Cluster A* (*n* = 3767): SIDD. Worse degree of glycemic control. *Cluster B* (*n* = 4810):SIRD. Greater baseline BMI. *Cluster C* (*n* = 4131): MOD. Greater baseline BMI and the lowest age of T2DM diagnosis. *Cluster D* (*n* = 7431):MARD. The highest age of T2DM diagnosis.
9.	Karpati et al. (2018) [15]	Israel	Retrospective cohort study	Clalit Health Services healthcare data warehouse	N = 85,783. participants had 3–7 years duration of type 2 diabetes. 60,423 from total number had valid HbA1c measures. The mean age of the study cohort was 63.6 years, 52.6% of the patients were female.	HbA1c tests, glucose tests, diagnoses, and diabetes medications were analyzed.	1. change in HbA1c values from t1 to t4 2. mean of the absolute first differences in HbA1c values 3. the ratio of the maximum absolute second difference to mean absolute first difference of HbA1c values	1. *Stable cluster* (*n* = 45,679) had 20.2% no treatment compared to the 8.0% in both the descending and ascending clusters; 2. *Descending cluster* (*n* = 6084) had the highest proportion of patients treated with insulin (and a possible additional non-insulin medication) (16.7% vs. 11.9% for the ascending cluster and 4.4% for the stable cluster, *p* < 0.001), had high proportion of micro- and macrovascular complications (28.0% and 16.4%) compared to the stable cluster; 3. *Ascending cluster* (*n* = 8660) had the highest proportion of patients being treated only with non-insulin hypoglycemic medication (79.5% in the ascending cluster vs. 75.3% for the descending and stable clusters, *p* < 0.001), showed frequent hypoglycemic events and high mortality (15.3%), had high proportion of micro- and macrovascular complications (28.8% and 15.4%) compared to the stable cluster; 4. *Undefined cluster* (*n* = 25,360) showed relatively low levels of micro and macrovascular complications, but had higher mortality rates (14.8%).
10.	Safai et al. (2018) [16]	Sweden	Observational retrospective study	Data from five cohorts: All New Diabetics in Scania (ANDIS), the Scania Diabetes Registry (SDR), All New Diabetics in Uppsala (ANDIU), Diabetes Registry Vaasa (DIREVA), and Malmö Diet and Cancer CardioVascular Arm (MDC-CVA).	N = 14,755. The results of 8980 patients from the ANDIS cohort were used for clustering. Patients from 5 databases with all types of diabetes.	Based on National Diabetes Registry	1. BMI 2. age at onset of diabetes 3. HOMA2-B 4. HbA1c 5. HOMA2-I 6. Presence or absence of GADA was included as a binary variable.	1. Autoimmune β-cell failure cluster (*n* = 65), characterized by patients with a positive GAD65 autoantibody titer. They also had the lowest TG level. 2. Insulin resistance with short disease duration cluster (*n* = 490), characterized by patients being diagnosed with type 2 diabetes relatively recently and having the highest HOMA2- β. 3. Non-autoimmune βcell failure cluster (*n* = 510), patients in sub-group 3 were the youngest at diabetes diagnosis but otherwise resembled sub-group 1 apart from the lack of positive GAD65 autoantibody titer. Increased risk for retinopathy. 4. Insulin resistance with long disease duration cluster (*n* = 727). Cluster 4 and 2 were very alike with a high age at diagnosis, similar BMI, better glycemic regulation, a relatively preserved β-cell function and at the same time a relatively high HOMA2-IR. The most important variable separating these two subgroups was the duration of diabetes. 5. Presence of metabolic syndrome cluster (*n* = 498), characterized by having the highest BMI compared to the other groups. It also consisted of those with the highest fasting glucose, HbA1c, C-peptide, HOMA2-IR and TG level.
11.	Zou et al. (2019) [17]	US and China	Cross-sectional population-based study	Data were taken from the 2007–2008 China National Diabetes and Metabolic Disorders Study (CNDMDS) and the 1988–94 National Health and Nutrition Examination Survey (NHANES III)	2316 participants from CNDMDS and 685 from NHANES III, (overall 3001)	WHO criteria	1. Age at diagnosis; 2.BMI; 3. HbA1c (or alternatively mean plasma glucose); 4.HOMA2-B 5.HOMA2-IR	*cluster 1* (MARD, 1045 (45.1%) of 2316 CNDMDS participants and 311 (45.4%) of 685 NHANES III) modest metabolic derangements in blood glucose, BMI, insulin resistance and β-cell function in both populations. *cluster 2* (MOD, 759 (32.7%) of 2316 CNDMDS participants and 222 (32.4%) of 685 NHANES III): highest BMI, yet average blood glucose, β-cell function, and insulin resistance in both populations; *cluster 3* (SIDD, 312 (13∙5%) of 2316 CNDMDS participants and 98 (14∙3%) of 685 NHANES III): had the lowest insulin secretion and highest blood glucose concentration; *cluster 4* (SIRD, 200 (8∙6%) of 2316 CNDMDS participants and 54 (7∙9%) of 685 NHANES III): had the highest insulin resistance and best beta cell function.
12.	Dennis et al. (2019) [18]	UK	Observational retrospective study	ADOPT trial, April, 2000, and June, 2002, followed up until June, 2006; For validation: RECORD cardiovascular outcomes trial, 2011 and 2003 followed up a minimum 5 years and a median 6 years	ADOPT trial (*n* = 4351, newly diagnosed T2DM patients aged 30–75 years); RECORD trail (*n* = 4447, 40–75 aged participants with established T2DM).	ADOPT trial: fasting plasma glucose 7–13 mmol/L, and no evidence of renal impairment; RECORD trial: HbA1c 7.0–9.0% (53–75 mmol/mol), BMI greater than 25.0 kg/m^2^ and no evidence of renal impairment	1. GADA levels; 2. Age at diagnosis; 3. BMI; 4. HbA1c; 5. HOMA-2b; 6. HOMA-IR.	Cluster 1 (SAID): 4.0%; Cluster 2 (SIDD): 20.0%; Cluster 3 (SIRD): 20.0%, had high BMI, HOMA-B and HOMA-IR, were at an older age; *Cluster 4* (MODD): 22.0%, had the highest BMI *Cluster 5* (MARD): 34.0%. In ADOPT trial clusters 1 (SAID), 2 (SIDD), and 4 (MOD) had higher rate of HbA1c progression, while only *cluster 4* (MOD) in RECORD trial. After adjustment to baseline UACR, time to albuminuria was shorter for *cluster 3* (SIRD) vs. *cluster 2* (SIDD) in ADOPT, but not RECORD.
13.	Li et al. (2015) [19]	USA	Cross-sectional study	Data from electronic medical records (EMRs) and genotype data (eMERGE)	From 11,210 genotyped outpatient cohort 2551 T2DM patients were included in the cluster analysis	ICD-9-CM diagnosis codes, laboratory tests (LONIC), prescribed medications (RxNorm)	Variables with at least 50% of patients who had the values, resulting in *73 variables* to perform the analysis were selected	Patients in *subtype 1* (762) were the youngest (59.76 ± 0.45 years) and were notable for features classically associated with T2DM, such as the highest BMI (33.07 ± 0.29 kg/m^2^) and highest serum glucose concentrations at point-of-care testing (POCT) (193.69 ± 11.45 mM). Although these patients had better kidney function compared to those in the other two subtypes. They were characterized by T2DM complications as diabetic nephropathy and diabetic retinopathy and ACE gene. Patients in *subtype 2* (617) had the lowest weight (85.17 ± 1.14 kg) compared with those in the other subtypes. *Subtype 2* was enriched for cancer malignancy and cardiovascular diseases. Patients in *subtype 3* (1096) had the highest SBP (135.7 ± 0.7 mmHg), serum chloride levels (102.03 ± 0.11 mEq/liter), and troponin I levels (0.36 ± 0.09 mg/liter) and were more often prescribed ARB/ACEI (62.96%) for the treatment of hypertension and statins (56.0%) for cholesterol reduction. They were associated most strongly with cardiovascular diseases, neurological diseases, allergies, and HIV infections and FHIT gene.
14.	Ahlqvist et al. (2017) [20]	Sweden	Observational retrospective study	Swedish ANDIS (All New Diabetics in Scania) cohort; For replication: The Scania Diabetes Registry (SDR); ANDIU (All new diabetics in Uppsala); DIREVA (Diabetes Registry Vaasa) MDC-CVA (Malmö Diet and Cancer)	ANDIS (N = 8980, aged 0–96 years, within a median of 40 days after diagnosis. ); SDR (N = 1466); ANDIU (N = 844); DIREVA (N = 3485); MDC-CVA (N = 3300)	Based on the National Diabetes Registry	1.GAD-antibodies 2.BMI 3.HbA1c 4.HOMA2-B 5.HOMA2-IR 6. Age at onset	*Cluster 1* (SAID, 6.4%); was characterized by early onset, relatively low BMI, poor metabolic control, insulin deficiency, and presence of GADA, frequent ketoacidosis (30.5%); *Cluster 2* (SIDD, 17.5%): was GADA negative but otherwise similar to SAID, frequent ketoacidosis (25.1%) and early signs of diabetic retinopathy; *Cluster 3* (SIRD, 15.3%): was characterized by insulin resistance (high HOMA2-IR) and high BMI, had the highest prevalence of non-alcoholic fatty liver disease and high risk for CKDs; *Cluster 4* (MODD, 21.6%): was characterized by obesity but not by insulin resistance; *Cluster 5* (MARD, 39.1%): were older, but showed, as *cluster 4*, only modest metabolic derangements.

BMI—body mass index. GADA—glutamic acid decarboxylase antibody; GAD65 antibody—glutamic acid decarboxylase 65 antibody; HbA1c—glycated hemoglobin; HOMA-2b—homoeostasis model assessment 2 estimates of β-cell function; HOMA-IR—homoeostasis model assessment 2 estimates of insulin resistance; FPG—fasting plasma glucose.

## Appendix B

**Table A2:** Assessment of CA methodology in the selected studies.

	Study (Author, Year)	Data Standardization	Methods of Clustering and Dimensionality Reduction	Methods for the Determination of the Number of Clusters (Direct or Statistical)	Clusters Validation on Independent Sample
1.	Ahlqvist et al. (2018) [7]	Yes	Hierarchical clustering, k-means for GADA-negative individuals	Silhouette width method	Yes
2.	Tanabe et al. (2020) [8]	No	Hierarchical clustering, k-means for GADA-negative individuals	Silhouette width method	No
3.	Zaharia et al. (2019) [9]	No	Hierarchical clustering	Silhouette width method	No
4.	Amato et al. (2016) [10]	Yes	Hierarchical clustering	Fixed number of clusters	No
5.	Arif et al. (2014) [11]	No	Agglomerative hierarchical clustering	Number of clusters were determined based on hierarchical clustering with Ward′s method	No
6.	Pes et al. (2016) [12]	No	PCA	Number of clusters were determined based on PCA (absolute factor loadings ≥ 0.4)	No
7.	Hammer et al. (2003) [13]	No	K-means, PCA	Number of clusters were determined based on PCA	No
8.	Kahkoska et al. (2020) [14]	Yes	Hierarchical clustering	Silhouette width method	No
9.	Karpati et al. (2018) [15]	No	K-means	“NbClust” algorithm that selected optimal method for determination of numbers of clusters	Yes
10.	Safai et al. (2018) [16]	Yes	k-means (Hartigan and Wong algorithm in R) and PCA for confirmation	Based on within the cluster sums of squares against the number of clusters	No
11.	Zou et al. (2019) [17]	No	K-means	Silhouette width method	No
12.	Dennis et al. (2019) [18]	Yes	K-means	Silhouette width method	Yes
13.	Li et al. (2015) [19]	Yes	Topology-based approach	Based on patient-patient network using cosine distance metric	Yes
14.	Ahlqvist et al. (2017) [20]	Yes	K-means	Silhouette width method	Yes
